# Editorial: Viral Infections in the Intensive Care Unit

**DOI:** 10.3389/fmed.2021.716824

**Published:** 2021-06-30

**Authors:** Alberto E. Maraolo, Aleksandra Barac, Olaf L. Cremer, David S. Y. Ong

**Affiliations:** ^1^First Division of Infectious Diseases, Cotugno Hospital, Naples, Italy; ^2^Clinic for Infectious and Tropical Diseases, Clinical Center of Serbia, Belgrade, Serbia; ^3^Faculty of Medicine, University of Belgrade, Belgrade, Serbia; ^4^Department of Intensive Care Medicine, University Medical Center Utrecht, Utrecht University, Utrecht, Netherlands; ^5^Department of Medical Microbiology and Infection Control, Franciscus Gasthuis & Vlietland, Rotterdam, Netherlands; ^6^Department of Epidemiology, Julius Center for Health Sciences and Primary Care, University Medical Center Utrecht, Utrecht University, Utrecht, Netherlands

**Keywords:** virus reactivation, intensive care unit, respiratory tract infection, herpesvirus, cytomegalovirus

With the emergence of the coronavirus disease 2019 (COVID-19) pandemic, respiratory viruses have regained attention as important infectious agents in a community setting (1, 2). In addition, it is increasingly being recognized that intensive care unit (ICU) patients are vulnerable to acquiring healthcare-associated infections that may in part be caused by viruses (3), and frequently experience reactivation of latent herpes viruses due to critical illness-induced impairment of cellular immunity (4, 5).

Making an adequate microbiological diagnosis is crucial in effective management of nosocomial infections. Repetitive diagnostic procedures (including various molecular tests as well as traditional bacterial and fungal culture techniques) are therefore common practice in ICUs worldwide. During these diagnostic workups, most clinicians tend to primarily focus their attention on bacterial microorganisms as the most likely causes for a suspected infection. As a result, many virus infections in ICU patients are likely to remain undetected, whereas their attributable burden may be underappreciated (6).

In this Frontiers research topic (https://www.frontiersin.org/research-topics/10696/viral-infections-in-the-intensive-care-unit#overview) five articles were selected that specifically address some of these issues: two articles related to the COVID-19 pandemic, one original research article on the relevance of respiratory syncytial virus (RSV), human metapneumovirus (hMPV), and human parainfluenza virus (hPIV) infections in hospitalized patients, one perspective on cytomegalovirus (CMV) reactivation during critical illness, and one comprehensive review regarding different epidemiological aspects of viral infections in ICU patients.

As COVID-19 is both more prevalent and possibly carries a worse prognosis in men (7), understanding gender-biases in SARS-CoV-2 infection is of paramount importance, not only from an epidemiological point of view but also in terms of clinical management. Chamekh and Casimir. argue in their opinion article that most data recorded in reports were not stratified by gender, which may result in gender-bias (https://doi.org/10.3389/fmed.2020.564117). The authors postulate that there is supporting evidence to include gender as an etiological variable, as several physiological factors are gender specific in infectious diseases, including sex hormones and sex-specific genetic architecture. As the severity of illness due to COVID-19 is related to excessive inflammation, the observation that men experience higher severity in comparison to women could be partially explained by the gender differences of the inflammatory response.

Sang et al. performed a single-center observational study on 20 patients with COVID-19 associated severe acute respiratory distress syndrome (ARDS) (https://doi.org/10.3389/fmed.2020.603943). The main study finding is that lung recruitability was very low during the late stage of disease. Furthermore, their findings suggest that individualized PEEP titration and use of prone positioning significantly improved PaO_2_, PaCO_2_, and static respiratory system compliance. Although similarities within ARDS regarding the pathophysiology and clinical presentation may exist, it remains crucial to also consider the heterogeneity within a clinical syndrome in order to optimize patients' treatment. At any rate, these findings need to be confirmed by prospective studies that include higher numbers of patients.

Chen et al. performed a retrospective observational study in 488 hospitalized immunocompetent adult patients with RSV, hMPV, or hPIV over a 9-year period (https://doi.org/10.3389/fmed.2020.574128). RSV caused more severe disease than hMPV and hPIV, and was the only independent predictor of 30-day mortality in multivariable analyses. Therefore, identification of RSV infection could be clinically relevant. With the development and possible emerging availability of antiviral treatment against RSV, further exploration of the role of RSV in immunocompetent adult patients is needed.

Many authors have contributed arguments for a tentative causal association between CMV reactivation (i.e., viremia or local reactivation in particular organs) and increased morbidity or mortality. However, final proof for pathogenicity of CMV reactivation in previously immunocompetent ICU patients is still lacking. Schildermans and De Vlieger. propose a strategy to resolve the question, which includes post-mortem investigations to evaluate the effect of viral replication on organ inflammation and function, the search for a threshold of viral load in plasma that correlates with clinical relevance, and the identification of biomarkers that could stratify patients at high risk for reactivation (https://doi.org/10.3389/fmed.2020.00188). After publication of this review, results of a long-awaited multicenter randomized clinical trial were published in early 2021 (8). In that trial, twice-weekly screening for CMV reactivation in whole blood followed by pre-emptive treatment with ganciclovir did not increase the number of ventilator-free days at day 60 in mechanically ventilated previously immunocompetent ICU patients. Unfortunately, the study was stopped prematurely due to a low inclusion rate and resulted in a total of no more than 39 ganciclovir and 37 placebo-treated controls. This study was part of a larger trial in which ICU patients were simultaneously monitored for HSV oropharyngeal reactivation and systemic CMV reactivation in blood. As HSV oropharyngeal reactivation occurs earlier in comparison to CMV reactivation, it is not surprising to see that the inclusion rate for the CMV part of the trial was low. Once acyclovir was started due to HSV reactivation, the patient became ineligible to be included in the CMV trial. In summary, this study was not able to provide evidence in favor of or against the use of pre-emptive ganciclovir in ICU patients. Therefore, a new and large phase III trial remains needed to answer whether pre-emptive ganciclovir treatment could be beneficial in previously immunocompetent ICU patients.

Finally, the research topic concludes with a comprehensive review by Fragkou et al. on the epidemiology, clinical features, diagnosis, pathogenesis, treatment and prevention of a number of viral pathogens relevant in an ICU setting, including SARS-CoV-2 (https://doi.org/10.3389/fmed.2021.575580). With the increasing availability of rapid molecular diagnostic techniques, it has become much easier to detect viral pathogens. However, these improved diagnostic capabilities by themselves do not help to accurately determine the true disease burden of viral infections in critically ill patients. Although no specific treatment is available for many viruses, we expect that this will likely change in the future. Many experimental antiviral and monoclonal antibody therapies are under investigation next to the exploration of immunomodulating interventions, which may also increase the clinical relevance of detection of virus infections in the future. The COVID-19 pandemic has accelerated these developments, and future studies should also include the assessment of which treatment strategies could favorably influence patient outcome.

## Author Contributions

All authors listed have made a substantial, direct and intellectual contribution to the work, and approved final version of the manuscript for submission.

## Conflict of Interest

The authors declare that the research was conducted in the absence of any commercial or financial relationships that could be construed as a potential conflict of interest.
